# Recurrent vasovagal syncope during a complicated tooth extraction: a case report

**DOI:** 10.1186/s12903-026-08108-w

**Published:** 2026-03-20

**Authors:** Samira Zouaghi

**Affiliations:** Dental Medicine Faculty of Monastir, University of Monastir, Monastir, 5019 Tunisia

**Keywords:** Case report, Vasovagal syncope, Dental extraction, Autonomic nervous system, Dental anxiety, Parasympathetic nervous system, Medical emergencies

## Abstract

Vasovagal syncope is the most common medical emergency encountered in dental practice and is typically benign. However, recurrence during the same operative session may present with escalating severity and misleading clinical recovery. This article reports a clinical observational case of recurrent vasovagal syncope occurring during a complicated mandibular third molar extraction in an anxious patient with a documented prior history of syncope. Two consecutive episodes occurred during the same session, the second associated with transient loss of consciousness. A subsequent appointment, managed through early prodromal recognition and conservative autonomic stabilization, allowed safe completion of treatment without syncope. This report emphasizes sustained nociceptive input, emotional stress, hypersalivation as a parasympathetic prodromal marker, and premature resumption of treatment as critical contributors to recurrence. A pathophysiological model of vasovagal recurrence and severity escalation adapted to dental practice is proposed, with practical implications for prevention and management.

**Supplementary Information:**

The online version contains supplementary material available at 10.1186/s12903-026-08108-w.

## Introduction

Medical emergencies in dental practice are predominantly neurovegetative in origin, with vasovagal syncope exceeding 60% of all incidents [1–5]. This neurally mediated reflex results from a sudden imbalance in autonomic control characterized by excessive parasympathetic activation and sympathetic withdrawal, leading to bradycardia, peripheral vasodilation, and cerebral hypoperfusion [6,7].

Dental procedures involving pain, anxiety, and prolonged duration—particularly complicated third molar extractions—constitute well-recognized high-risk scenarios [8,9]. However, the literature provides limited detailed accounts of recurrent vasovagal episodes during a single dental session, especially in patients with documented previous vasovagal history.

The present case report addresses this gap by documenting recurrent vasovagal syncope with escalating severity in a patient with prior syncope history during a previous simple extraction. We aim to: (i) detail the chronological evolution of recurrent episodes during a single dental procedure, (ii) examine the pathophysiological mechanisms underlying recurrence and severity escalation, and (iii) provide practical guidance for dental practitioners for prevention and management.

## Case report

### Patient history and initial presentation

A 33-year-old female patient with no relevant medical history presented for emergency management of severe dental pain. Clinical and radiographic examination confirmed the diagnosis of symptomatic irreversible pulpitis affecting the right mandibular third molar, for which extraction was indicated.

### Relevant dental and vasovagal history

During the consultation, the patient asked numerous questions about the procedural details. Upon inquiry, she disclosed that this would be her second dental extraction. Her first extraction involved a maxillary third molar, which she described as technically straightforward but emotionally stressful, being her first experience with dental extraction. Notably, she had experienced a vasovagal episode during that previous uncomplicated extraction, attributed to her anxiety regarding the novel procedure.

Prior to the current extraction, the patient had been informed that the mandibular third molar extraction would be significantly more complex than the previous one. This anticipatory knowledge contributed to marked pre-procedural anxiety.

### First appointment: complicated extraction and recurrent vasovagal episodes

From the outset, the patient exhibited marked procedural anxiety. Despite correct anesthetic technique, profound analgesia was difficult to achieve, requiring multiple administrations of local anesthetic. Throughout the procedure, pronounced hypersalivation was observed, suggesting parasympathetic predominance.

The extraction proved technically challenging. Crown fracture occurred during instrumentation, leaving two of three roots retained, thereby prolonging the operative time and increasing procedural stress.

#### First vasovagal episode

During attempts to remove the remaining roots, the patient experienced a first vasovagal episode without loss of consciousness, characterized by malaise, pallor and nausea. The procedure was immediately interrupted, all instruments were removed from the oral cavity, and the patient was reassured and positioned supine with lower limb elevation. Clinical recovery was rapid and apparently complete.

#### Second vasovagal episode

Approximately ten minutes after resuming the procedure, the patient developed a second vasovagal episode, this time with transient loss of consciousness, prompting definitive cessation of the procedure and implementation of appropriate emergency management protocols. A subsequent appointment was scheduled to complete the extraction.

### Follow-up appointment: successful completion with prodromal management

At the second appointment scheduled to remove the remaining roots, the patient again exhibited anxiety related to the previous experience. During the procedure completion, she developed prodromal symptoms consistent with an impending vasovagal episode. The procedure was immediately interrupted, and the patient was placed in a supine position. With extended observation and supportive management, the extraction was successfully completed without syncope. The patient was monitored until complete stabilization before discharge and she was advised to seek medical evaluation in case of recurrence.

Timeline of clinical events:

- Local anesthesia administration
- Initiation of extraction procedure
- Onset of prodromal symptoms (dizziness, hypersalivation)
- First syncopal episode
- Partial clinical recovery
- Recurrence during continuation of the procedure
- Procedure interruption and patient stabilization

## Discussion

### Epidemiology of vasovagal syncope in dental practice

Syncope represents the leading medical emergency in dental settings, with recent multicenter studies confirming that vasovagal episodes remain the most frequent complication [1–5]. The incidence is particularly elevated during surgical procedures and in patients with high anxiety levels [9].

A recent systematic review and meta-analysis reported a high global prevalence of vasovagal syncope in the general population [10]. In dental contexts, the risk is amplified by procedure-specific triggers including pain, anxiety, and the supine-to-upright postural transition [8,9].

### Pathophysiological mechanisms

Vasovagal syncope results from a sudden imbalance in autonomic control, characterized by excessive parasympathetic activation and sympathetic withdrawal. The resulting bradycardia, peripheral vasodilation, reduced venous return, and hypotension lead to transient cerebral hypoperfusion [7].

#### Autonomic reflex arc[7]

Vasovagal syncope is mediated by a complex reflex arc involving central autonomic networks, particularly the medullary vasomotor center, baroreceptors, and cardiopulmonary mechanoreceptors. The reflex cascade includes:

1. Trigger activation: Nociceptive input, emotional stress, or fear activates higher cortical and limbic centers.
2. Parasympathetic surge: Excessive vagal outflow via the nucleus tractus solitarius.
3. Sympathetic withdrawal: Reduced peripheral vascular resistance.
4. Cardiovascular collapse: Bradycardia, hypotension, and reduced cardiac output.
5. Cerebral hypoperfusion: Loss of consciousness when cerebral blood flow falls below critical threshold.

#### Synergistic triggers [9, 11]

Acute pain activates nociceptive pathways projecting to the anterior cingulate cortex and insula, regions integral to autonomic regulation. When combined with anxiety—which independently increases vagal tone through limbic activation—the threshold for vasovagal reflex triggering is substantially lowered.

In the present case, several synergistic triggers were present:

- Previous vasovagal history creating anticipatory anxiety.
- Inadequate analgesia maintaining sustained nociceptive stimulation and requiring repeated anesthetic administration.
- Prolonged procedure duration due to technical complications.
- Psychological distress from intraoperative crown fracture.

#### Hypersalivation as a parasympathetic prodromal marker [12]

Hypersalivation reflects direct parasympathetic stimulation of salivary glands via the facial (VII) and glossopharyngeal (IX) cranial nerves. This phenomenon serves as a reliable prodromal indicator of impending vasovagal syncope, often appearing before hemodynamic collapse. Its persistence after the apparent initial recovery of the first episode suggests an ongoing autonomic instability and should be interpreted as a warning sign of imminent recurrence.

### Previous vasovagal history as a major risk factor [9, 10, 13]

Patients with documented previous vasovagal episodes demonstrate a significantly elevated recurrence risk, with likelihood of repeat events when exposed to similar triggers. Several mechanisms explain this vulnerability:

1. Autonomic sensitization: Previous episodes may create lasting alterations in baroreflex sensitivity.
2. Anticipatory anxiety: Knowledge of previous syncope generates fear-avoidance behaviors and heightened vigilance, paradoxically increasing autonomic reactivity.
3. Psychological conditioning: Classical conditioning may link dental environments with vasovagal responses.

In this case, the patient’s disclosure of syncope during her first, technically simple extraction is particularly significant. Despite the procedural ease, the novelty and associated anxiety were sufficient triggers—a pattern indicating high intrinsic susceptibility to vasovagal responses. The anticipatory anxiety generated by foreknowledge of increased complexity in the current procedure likely primed her autonomic system for exaggerated responses.

### Original model of vasovagal recurrence and severity escalation

The progression from a vasovagal episode without loss of consciousness to frank syncope within the same session in our patient represents a critical clinical phenomenon inadequately addressed in dental literature.

We propose a three-phase model of escalating autonomic dysfunction inspired by classical descriptions of neurally mediated syncope and adapted to dental practice:

#### Phase 1: Trigger accumulation

Sustained pain, anxiety, and procedural stress progressively increase vagal tone. Inadequate anesthesia requiring multiple injections perpetuates nociceptive input, preventing autonomic stabilization

#### Phase 2: Incomplete autonomic reset

Although the patient demonstrates rapid clinical recovery after the first episode, apparent recovery does not equate to complete autonomic stabilization. Studies using continuous autonomic monitoring demonstrate that parasympathetic dominance may persist for 15–30 min despite normal vital signs. Simultaneously, sympathetic compensatory mechanisms become exhausted through repeated activation. 

#### Phase 3: Premature re-exposure and escalation

Resumption of the dental act within this vulnerable window reintroduces nociceptive and emotional stimuli to an autonomically unstable system. The exhausted sympathetic response cannot adequately compensate, resulting in more severe parasympathetic dominance and progression to syncope with loss of consciousness.

The proposed three-phase framework should be interpreted as a conceptual application of established autonomic physiology to the observed clinical sequence rather than as a direct physiological demonstration, as no continuous autonomic or hemodynamic monitoring was performed.

### Clinical recognition: prodromal signs

Alternative causes of transient loss of consciousness were clinically considered. The presence of clear prodromal autonomic symptoms, rapid spontaneous recovery, and the procedural trigger strongly supported a vasovagal mechanism.

From a differential diagnostic perspective, distinguishing vasovagal syncope from cardiac syncope is essential in dental practice. Vasovagal syncope is typically preceded by prodromal symptoms such as nausea, diaphoresis, pallor, dizziness, and hypersalivation. In contrast, cardiac syncope is often abrupt, may occur without warning, and is frequently associated with underlying arrhythmias or structural heart disease. The absence of exertional triggers and the presence of clear prodromal autonomic symptoms in the present case strongly support a vasovagal mechanism.

Early recognition of prodromal symptoms is essential for prevention. The classical prodrome includes [13]:

- Cardiovascular: Palpitations progressing to bradycardia
- Autonomic: Diaphoresis, pallor, hypersalivation
- Neurological: Lightheadedness, visual disturbances (graying out, tunnel vision), tinnitus
- Gastrointestinal: Nausea, abdominal discomfort
- Subjective: Sense of impending doom, feeling of warmth

The patient in this case demonstrated prominent nausea, hypersalivation and malaise during both the first and second vasovagal episodes, and notably, prodromal symptoms during the follow-up appointment. The successful management at the second appointment, through immediate recognition and intervention, demonstrates the critical value of vigilance in high-risk patients.

### Evidence-based management [14]

First-line management of vasovagal syncope primarily relies on conservative strategies including adequate hydration, avoidance of known triggers, recognition of prodromal symptoms and physical counterpressure maneuvers such as leg crossing, squatting, or limb contraction to maintain cerebral perfusion.

#### Immediate management of acute episode

Current recommendations emphasize [14]:

1. Immediate interruption of the procedure at the first prodromal signs.
2. Supine positioning with legs elevation to enhance venous return.
3. Airway management: Ensure patency; remove all materials from the oral cavity.
4. Vital signs monitoring: Pulse, blood pressure, oxygen saturation until complete autonomic stabilization.
5. Reassurance: Verbal comfort to reduce anxiety.
6. Observation: Minimum 15–20 min before considering vertical positioning.

#### Critical decision point: treatment resumption

The present case provides compelling evidence supporting current recommendations against resuming treatment during the same session after a vasovagal episode. Key considerations include:

- Autonomic instability persists despite apparent clinical recovery.
- Risk of recurrence increases with each episode.
- Severity may escalate with repeated episodes.
- Prodromal signs (particularly hypersalivation) indicate ongoing vulnerability.

Recommended approach:

- If initial recovery is complete and procedure is nearly finished, cautious completion may be considered with continuous monitoring.
- If significant work remains, defer to subsequent appointment with enhanced anxiety management.
- Never resume if prodromal signs persist (hypersalivation, nausea, pallor).

#### Management for subsequent appointments

For patients with vasovagal history, subsequent appointments require enhanced protocols :

Pre-operative measures :

- Detailed anxiety assessment and counseling
- Consider anxiolytic premedication (e.g., oral benzodiazepines if medically appropriate)
- Morning appointments when patient is well-rested
- Adequate hydration and food intake prior to appointment
- Avoid prolonged waiting periods

Intra-operative measures:

- Optimal positioning: semi-reclined rather than fully supine initially
- Gradual position changes
- Profound anesthesia achieved before initiating invasive procedures
- Intermittent breaks during prolonged procedures
- Continuous verbal communication and reassurance
- Monitoring for prodromal signs
- Availability of emergency equipment and trained personnel

Post-operative measures:

- Gradual return to upright position over several minutes
- Extended observation after procedure completion before discharge
- Clear discharge instructions regarding delayed symptoms

Patients presenting with recurrent syncopal episodes or inadequate response to conservative measures should be referred for further medical evaluation, particularly when additional dental procedures are planned and pharmacological therapies (midodrine, fludrocortisone) [15] may be advised for these patients.

In refractory cases, interventional approaches like cardioneuroablation [16] may be considered by medical specialists.

### Prognosis and long-term outcomes

Vasovagal syncope, while benign in terms of mortality risk, significantly impacts quality of life and healthcare-seeking behavior [11]. Patients with recurrent episodes may develop:

- Dental avoidance: Leading to deteriorating oral health.
- Injury from falls: If syncope occurs unexpectedly.
- Psychological distress: Anxiety about future episodes.

However, with appropriate recognition, management, and preventive strategies, the vast majority of patients can undergo necessary dental procedures safely.

### Limitations

This report has limitations inherent to single case studies. In addition, objective physiological parameters such as blood pressure, heart rate, and oxygen saturation were not systematically recorded during the syncopal episodes due to the acute clinical context. This lack of hemodynamic and autonomic monitoring limits objective confirmation of the proposed pathophysiological interpretation which remains conceptual and based on the established physiology of the observed clinical sequence rather than direct hemodynamic measurements.

## Conclusions

This case report illustrates that recurrent vasovagal syncope during dental treatment represents a dynamic and potentially escalating autonomic disturbance, particularly in patients with previous vasovagal history. The patient’s disclosure of syncope during a previous uncomplicated extraction should have been recognized as a major risk factor warranting enhanced preventive measures from the outset.

The progression from non-syncopal vasovagal symptoms to frank syncope within the same session, followed by successful prodromal management during a subsequent appointment, suggests both the risks of premature treatment resumption and the effectiveness of conservative, vigilant management strategies.

Dental practitioners must actively screen for previous vasovagal history, recognize that anticipatory anxiety in susceptible patients significantly amplifies risk, maintain vigilance for prodromal signs—particularly hypersalivation—and adhere strictly to conservative protocols after any vasovagal episode. The principle that apparent recovery does not equal autonomic stability should guide clinical decision-making.

With appropriate anxiety management, early prodromal recognition, and evidence-based interventions, the vast majority of patients with vasovagal history can safely undergo necessary dental procedures, as demonstrated by the successful completion of treatment at the second appointment in this case.

## Supplementary Information

Supplementary Material 1.

## Data Availability

All data generated or analyzed during this study are included in this published article.
