# Supplementary material for: Recurrent vasovagal syncope during a complicated tooth extraction: a case report
Source: BMC Oral Health. 2026 Mar 20;26:758. doi: 10.1186/s12903-026-08108-w (PMC13126704; doi:10.1186/s12903-026-08108-w)
Supplement: Supplementary file 1 — Supplementary Material 1. [file 12903_2026_8108_MOESM1_ESM.docx]

#
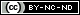

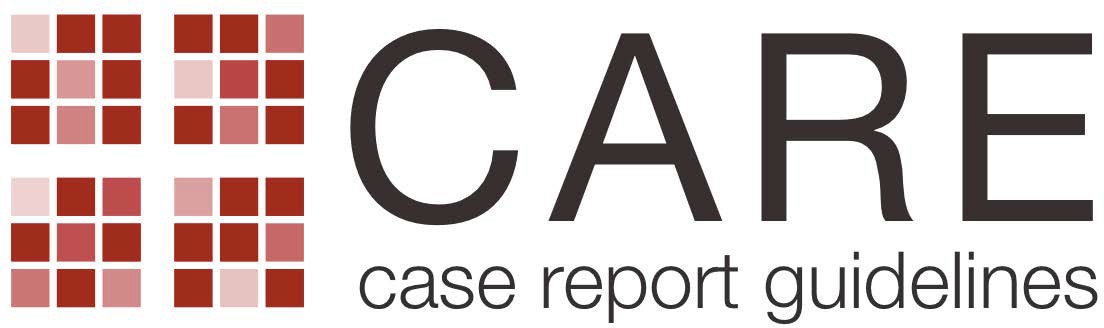
CARE Checklist of information to include when writing a case report

**Topic Item Checklist item description Reported on Line**

**Title 1** The diagnosis or intervention of primary focus followed by the words “case report” . . . . . . . . . . . . . . . . . . Line 1/2

**Key Words 2** 2 to 5 key words that identify diagnoses or interventions in this case report, including "case report" Lines 24/25

**Abstract**

**(no references)**

**3a** Introduction: What is unique about this case and what does it add to the scientific literature? Lines 19-21

**3b** Main symptoms and/or important clinical findings . . . . . . . . . . . . . . . . . . . . . . . . . . . . . . . . . . . . . . . . . . . . . . . . . . . Lines 13-19

**3c** The main diagnoses, therapeutic interventions, and outcomes Lines 13-19

**3d** Conclusion—What is the main “take-away” lesson(s) from this case? Lines 19-23

**Introduction 4** One or two paragraphs summarizing why this case is unique (**may include references**) Lines 33-36

**Patient Information 5a** De-identified patient specific information Line 45

**5b** Primary concerns and symptoms of the patient Lines 46-48

**5c** Medical, family, and psycho-social history including relevant genetic information Line 45

**5d** Relevant past interventions with outcomes Lines 49-55

**Clinical Findings**

**Timeline**

**Diagnostic Assessment**

**Therapeutic Intervention**

**Follow-up and Outcomes**

1. Describe significant physical examination (PE) and important clinical findings Lines 59-86
2. Historical and current information from this episode of care organized as a timeline Lines 49-86

**8a** Diagnostic testing (such as PE, laboratory testing, imaging, surveys). N/A

**8b** Diagnostic challenges (such as access to testing, financial, or cultural) N/A

**8c** Diagnosis (including other diagnoses considered) Lines 68-78

**8d** Prognosis (such as staging in oncology) where applicable Lines 79-86

**9a** Types of therapeutic intervention (such as pharmacologic, surgical, preventive, self-care) . . . . . . . . . . . . . . . . . Lines 70-72

**9b** Administration of therapeutic intervention (such as dosage, strength, duration) N/A

**9c** Changes in therapeutic intervention (with rationale) N/A

**10a** Clinician and patient-assessed outcomes (if available) Lines 179-181

**10b** Important follow-up diagnostic and other test results N/A

**10c** Intervention adherence and tolerability (How was this assessed?) N/A

**10d** Adverse and unanticipated events N/A

**Discussion 11a** A scientific discussion of the strengths AND limitations associated with this case report N/A

**11b** Discussion of the relevant medical literature **with references** Lines 89-112

**11c** The scientific rationale for any conclusions (including assessment of possible causes) Lines 97-123

**11d** The primary “take-away” lessons of this case report (without references) in a one paragraph conclusion Lines 246-250

**Patient Perspective 12** The patient should share their perspective in one to two paragraphs on the treatment(s) they received . . . . N/A

**Informed Consent 13** Did the patient give informed consent? Please provide if requested . . . . . . . . . . . . . . . . . . . . . . . . . . . . . . . . . . . . . . **Yes
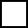
** **No
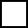
**
